# A set of aspartyl protease-deficient strains for improved expression of heterologous proteins in *Kluyveromyces lactis*

**DOI:** 10.1111/j.1567-1364.2010.00703.x

**Published:** 2010-12-17

**Authors:** Mehul B Ganatra, Saulius Vainauskas, Julia M Hong, Troy E Taylor, John-Paul M Denson, Dominic Esposito, Jeremiah D Read, Hana Schmeisser, Kathryn C Zoon, James L Hartley, Christopher H Taron

**Affiliations:** 1 Division of Gene Expression, New England BiolabsIpswich, MA, USA; 2 Protein Expression Laboratory, Advanced Technology Program, SAIC-Frederick Inc., National Cancer Institute at FrederickFrederick, MD, USA; 3 The Cytokine Biology Section, The Division of Intramural Research, NIAID, NIHBethesda, MD, USA

**Keywords:** aspartic protease, protein expression, protein degradation, yapsin, *Kluyveromyces lactis*

## Abstract

Secretion of recombinant proteins is a common strategy for heterologous protein expression using the yeast *Kluyveromyces lactis*. However, a common problem is degradation of a target recombinant protein by secretory pathway aspartyl proteases. In this study, we identified five putative pfam00026 aspartyl proteases encoded by the *K. lactis* genome. A set of selectable marker-free protease deletion mutants was constructed in the prototrophic *K. lactis* GG799 industrial expression strain background using a PCR-based dominant marker recycling method based on the *Aspergillus nidulans* acetamidase gene (*amdS*). Each mutant was assessed for its secretion of protease activity, its health and growth characteristics, and its ability to efficiently produce heterologous proteins. In particular, despite having a longer lag phase and slower growth compared with the other mutants, a Δ*yps1* mutant demonstrated marked improvement in both the yield and the quality of *Gaussia princeps* luciferase and the human chimeric interferon Hy3, two proteins that experienced significant proteolysis when secreted from the wild-type parent strain.

## Introduction

The yeast *Kluyveromyces lactis* has been used as a host for the production of heterologous proteins at both laboratory and industrial scale for over two decades (van den Berg *et al.*, 1990; van Ooyen *et al.*, 2006). A common strategy for producing heterologous proteins in *K. lactis* is to target their export from the cell via the secretory pathway. This approach gives heterologous proteins access to both the endoplasmic reticulum chaperones and the glycosylation machinery required for the correct folding and export of many extracellular eukaryotic proteins.

A factor that limits both the overall yield and the quality of some secreted heterologous proteins is their degradation by endogenous host proteases. In yeasts, secretory pathway proteases are found in the vacuole, the Golgi, the plasma membrane, the cell wall or are actively secreted into the extracellular space. While most vacuolar proteases are zymogens and do not become active until they reach the vacuole, other secretory proteases such as Bar1p (Ballensiefen & Schmitt, 1997) and yapsins become active during their export or at the plasma membrane, increasing the potential for detrimental processing of secreted heterologous proteins. Indeed, in certain yapsin deletion strains of *Saccharomyces cerevisiae* and *Pichia pastoris*, reduced proteolysis of secreted recombinant proteins has been reported (Rourke *et al.*, 1997; Copley *et al.*, 1998; Kang *et al.*, 1998; Kerry-Williams *et al.*, 1998; Bourbonnais *et al.*, 2000; Egel-Mitani *et al.*, 2000; Werten & de Wolf, 2005; Yao *et al.*, 2009). Additionally, proteases may populate the growth medium during cultivation of yeast cells due to shedding of glycosylphosphatidylinositol-anchored proteases from the plasma membrane or cell wall, or from cell lysis (Ray *et al.*, 1992; Ogrydziak, 1993; Aoki *et al.*, 1994; Sinha *et al.*, 2004).

We recently cataloged the proteins comprising the secreted proteome of the industrial *K. lactis* expression host strain GG799 propagated at high cell density in a bioreactor (Swaim *et al.*, 2008; Madinger *et al.*, 2009). In those studies, members of a eukaryotic secretory aspartyl protease family (pfam00026) that includes cathepsin D, pepsin, renin, penicillopepsin and fungal yapsins were observed in several growth conditions. In the present study, we examined the *K. lactis* genome to identify genes encoding deduced proteins with homology to pfam00026 aspartyl proteases. Five putative pfam00026 proteins (Yps1p, Yps7p, Pep4p, Bar1p and the putative protein encoded by locus KLLA0D01507g) were found.

Several homologs of the five *K. lactis* pfam00026 proteins have been functionally characterized in other yeasts. In *S. cerevisiae*, Bar1p is a periplasmic protease that mediates pheromone degradation and promotes mating (Chan & Otte, 1982; MacKay *et al.*, 1988; Egel-Mitani *et al.*, 1990; Ballensiefen & Schmitt, 1997). Pep4p is a soluble vacuolar protease (proteinase A) required for the post-translational precursor maturation of vacuolar proteinases that are important for protein turnover after oxidative damage (Woolford *et al.*, 1986; Rupp & Wolf, 1995; Marques *et al.*, 2006). *Kluyveromyces lactis* Yps1 and Yps7 are yapsin family proteases that are putatively attached to the plasma membrane or cell wall via a glycosylphosphatidylinositol anchor. Additionally, the putative *K. lactis* protease encoded by KLLA0D01507g is most similar to the Yps6p yapsin family protease of *S. cerevisiase*. In *S. cerevisiae* and *Candida glabrata*, yapsins play a critical role in maintaining the integrity of the cell wall (Krysan *et al.*, 2005; Kaur *et al.*, 2007) and differ from other aspartyl proteases by cleaving proteins and peptides on the C-terminal side of monobasic residues (typically Lys or Arg) instead of hydrophobic residues (Komano *et al.*, 1999; Olsen *et al.*, 1999).

We report here the construction of a set of five pfam00026 aspartyl protease deletion mutants in the *K. lactis* GG799 industrial expression strain using a PCR-based selectable marker-recycling gene deletion strategy. Each mutant strain was assessed for its growth characteristics, its total secreted proteolytic activity and its ability to be used for expression of secreted recombinant proteins. We demonstrate that certain mutant strains improve the quality and yield of *Gaussia princeps* luciferase (Verhaegent & Christopoulos, 2002) and chimeric human interferon Hy3 (Hu *et al.*, 1999), two secreted heterologous proteins that experience proteolysis when expressed in the *K. lactis* GG799 parent strain.

## Materials and methods

### Yeast strains, media and culturing conditions

All mutant strains described in this study were created in the *K. lactis* GG799 expression strain background (Colussi & Taron, 2005). *Kluyveromyces lactis* strains were routinely grown in YPGal medium (1% yeast extract, 2% peptone, 2% galactose, optionally containing 2% agar for solid medium), YPGlu medium (1% yeast extract, 2% peptone and 2% glucose, ±2% agar) or YPGly medium (1% yeast extract, 2% peptone and 2% glycerol, ±2% agar) at 30 °C for 2–3 days. Nitrogen-free yeast carbon base (YCB) medium and acetamide were from New England Biolabs (Ipswich, MA). G418 was from Sigma-Aldrich (St. Louis, MO) and used in YPGal medium at a final concentration of 200 μg mL^−1^. In all experiments, samples of spent culture medium (SCM) were prepared by clearing cells from aliquots of liquid cultures grown to saturation by centrifugation at 4000 ***g*** for 10 min.

### PCR

All oligonucleotide primers used for PCR-based assembly of gene disruption DNA fragments and for whole-cell PCR identification of chromosomal integration events are presented in Supporting Information, Table S1.

**Fig. 1:**
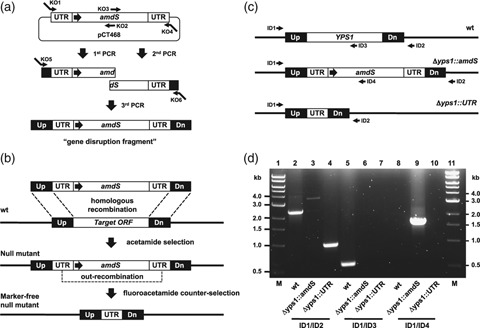
Gene deletion and *amdS* marker recycling strategy. (a) A linear gene disruption DNA fragment was assembled by three rounds of PCR. The fragment contained the *Aspergillus nidulans amdS* gene flanked by directly repeating 300-bp segments of its native 3′ untranslated region (UTR) and typically 160–250 bp of DNA homologous to regions upstream (Up) and downstream (Dn) of the target chromosomal locus. Expression of *amdS* was driven by the *Saccharomyces cerevisiae ADH1* promoter (large horizontal arrow). (b) Upon its introduction into *Kluyveromyces lactis* cells, transplacement of the disruption fragment occurs at the target locus by homologous recombination resulting in gene deletion. Integrants are selected by growth on nitrogen-free medium containing acetamide. Subsequent out-recombination of the *amdS* marker occurs in the absence of selective pressure and *amdS*^−^ null mutants are isolated by growth on counterselection medium containing fluoroacetamide. (c) PCR using a single forward primer (ID1) and various strategically positioned reverse primers (ID2-4) are used to assess the integrity of a modified locus. (d) An agarose gel showing an example of genomic PCR analysis of strains harboring Δ*yps1*∷*amdS* and marker-free Δ*yps1*∷*UTR* null alleles.

DNA constructs for disruption of *K. lactis* genes encoding putative pfam00026 proteases were assembled using a multistep ‘PCR-knitting’ strategy shown in Fig. 1a. The PCR template vector pCT468 contained an expression cassette consisting of the *Aspergillus nidulans* acetamidase gene (*amdS*) cloned downstream of the *S. cerevisiae* alcohol dehydrogenase (*ADH1*) promoter. This cassette was flanked on both sides by 300 bp directly repeating DNA sequences comprising the *amdS* gene's native 3′ untranslated region (UTR). The entire cassette was assembled by gene synthesis and cloned into the KpnI and HindIII sites of pUC57 (GenScript USA, Piscataway, NJ) and its sequence is available from GenBank (#HM015509). In the PCR knitting strategy, two halves of a gene disruption fragment were amplified by PCR using primer pairs KO1/KO2 and KO3/KO4. Primers KO1 and KO4 each contained tails homologous to the 5′ and 3′ ends of the target chromosomal integration site, respectively. Primer tail lengths were from 80- to 125-bp long depending on the individual gene being deleted (precise tail lengths are noted in Table S1). Additionally, the 3′ end of the ‘left’ amplicon and the 5′ end of the ‘right’ amplicon had overlapping complimentary regions. Amplification was performed in 1 × Phusion HF buffer containing 2% dimethyl sulfoxide, 1 mM MgCl_2_, 200 μM dNTPs, 0.5 μM of each primer, 125 ng pCT468 and 0.04 U Phusion™ DNA polymerase (New England Biolabs) in a total reaction volume of 100 μL. Thermocycling consisted of incubation at 98 °C for 40 s followed by 35 cycles of successive incubations at 98 °C for 10 s and 72 °C for 2 min. After thermocycling, a final extension was performed at 72 °C for 8 min.

The two halves of the disruption fragment were ‘knitted’ together by an additional round of PCR. In this strategy, complimentary regions in the left and right amplicons annealed to each other and were extended by the polymerase to form a full-length disruption fragment template that was subsequently amplified by extension of primers KO5 and KO6. Primers KO5 and KO6 also contained tails of additional chromosomal targeting sequence (∼80–125 bp) that elongate the targeting sequence first introduced by primers KO1 and KO4. Thus, final amplified disruption fragments contained from 160- to 250-bp chromosomal targeting sequence on each end, depending upon the specific primer lengths used for each gene. The reaction conditions for knitting PCR were identical to those above, with the exception that 500 ng each of the left and right amplicons was used as template, and an extension of 3 min at 72 °C was used during thermocyling.

Whole-cell PCR was used to assess the integrity of a target chromosomal locus either after integrative transformation of cells with a disruption fragment or after out-recombination of the *amdS* marker, using primer pairs that direct amplification of specific diagnostic DNA fragments (Fig. 1c and d). Candidate *K. lactis* strains were patched onto YCB agar plates containing 5 mM acetamide and incubated overnight at 30 °C. A sterile pipette tip was used to scrape approximately 1 mm^2^ of cells into 25 μL of a 1 mg zymolyase mL^−1^ solution in 30 mM sodium phosphate (the Associates of Cape Cod, East Falmouth, MA). The cells were incubated at 25 °C for 1 h to allow for cell wall digestion, after which the cells were lysed and DNA was denatured by incubation at 98 °C for 10 min. The temperature was lowered to 80 °C and 75 μL 1 × ThermoPol buffer containing 200 μM dNTPs, 0.8 μM of each ID primer and 5 U Taq DNA polymerase (New England Biolabs). Thermocycling consisted of 30 cycles of successive incubations at 95 °C for 30 s, 50 °C for 30 s and 72 °C for 2 min. After cycling, a final extension was performed at 72 °C for 10 min.

### Construction of protease-deficient strains

After its assembly by PCR, 2 μg of a gene disruption DNA fragment was introduced into *K. lactis* GG799 competent cells as described by the manufacturer (New England Biolabs) followed by selection of transformants by growth on YCB agar medium supplemented with 5 mM acetamide for no more than 3 days at 30 °C. Successful disruption of a target chromosomal locus was assessed by whole-cell PCR (see previous section).

To recycle the *amdS* marker, a strain harboring an *amdS*^+^ disrupted target allele was grown in the absence of selection on YPD agar to permit recombination between the directly repeating UTR regions that flank the *amdS* gene (Fig. 1b). Null mutants lacking the *amdS* gene were then isolated by three rounds of restreaking on YCB agar supplemented with 10 mM fluoroacetamide (Sigma-Aldrich) and 0.1% (w/v) ammonium sulfate, and incubation for 2–3 days at 30 °C.

### Analysis of cell health and growth

Growth curves and measurement of cellular biomass produced during culturing of null mutant strains was performed by growing strains in 250-mL shake-flasks containing 100 mL YPGal medium at 30 °C for 72 h. Cell growth was measured by OD_600 nm_ in triplicate in an Ultraspec 2100 Pro spectrophotometer (GE Healthcare, Piscataway, NJ). After 12, 24, 48 and 72 h of growth, 10 mL of each culture were removed and cells were pelleted by centrifugation at 4000 ***g*** for 10 min. Cell pellets were washed once in water to remove medium components and dried in disposable preweighed aluminum pans (ThermoFisher Scientific, Waltham, MA). The dry cell mass (g L^−1^) was calculated.

Null mutant strains were examined for defects in cell wall integrity by assessing their growth compared with wild-type GG799 cells on YPD agar medium supplemented with the cell wall-perturbing compounds Congo Red (10 μg mL^−1^; Sigma-Aldrich) or Calcofluor white (100 μg mL^−1^; Sigma-Aldrich) at 30 °C for 2–3 days (Kopecká & Gabriel, 1992; Ram *et al.*, 1994).

### Protease assays

Protease activity in SCM of each mutant strain was measured by two methods. In each assay, protease activity was determined directly from SCM prepared from cells grown to saturation. Measurements were normalized to each culture's final OD_600 nm_ to account for any slight differences in cell growth. All reactions were performed in triplicate.

Total protease activity in SCM was measured using IRDye 800RS casein (LI-COR Biosciences, Lincoln, NE) as a fluorogenic substrate. Reactions were carried out by incubating 50 μL of SCM with 19 pmol of the IRDye 800RS casein substrate (0.126 μM final concentration) in 150 μL of 0.05 M Tris-HCl buffer (pH 7.2) containing 0.05% Tween 20 (v/v) and 0.01% sodium azide (w/v) at 30 °C for 20 h in the dark. The fluorescence intensity of reactions was measured using the 800-nm channel of an Odyssey® Infrared Imaging System (LI-COR).

Activity of subtilisin-type and yapsin-like proteases in SCM was assayed using the chromogenic substrate Z-Tyr-Lys-Arg-pNA (Bachem, Switzerland). The reaction was carried out by incubating 50 μL of SCM with 0.15 mM substrate in 50 mM Tris-HCl (pH 7.2) containing 1 mM CaCl_2_ in a total volume of 100 μL at 30 °C for 24 h. The reaction was terminated by the addition of EDTA to a final concentration of 10 mM. Liberation of *p*-nitroanilide (pNA) was measured at 405 nm in a SpectraMax M5 spectrophotometer (Molecular Devices, Sunnyvale, CA).

### *Gaussia princeps* luciferase expression and assay

Secreted expression of *G. princeps* luciferase (Gluc) in *K. lactis* GG799 cells has been reported previously (Read *et al.*, 2007). Briefly, DNA encoding the *G. princeps* luciferase ORF was cloned downstream of DNA encoding the *K. lactis*α-mating factor secretion leader in the integrative *K. lactis* expression vector pGBN19 to yield pGBN19-Gluc. In the present study, 2 μg of pGBN19-Gluc was linearized by SacII digestion and introduced into each of the protease null mutant strains using a lithium acetate transformation procedure (Read *et al.*, 2007), after which transformants were selected by growth on YPGal medium containing 200 μg G418 mL^−1^. Strains harboring a single-copy insertion of pGBN19-Gluc into the *LAC4* locus of the *K. lactis* chromosome were identified by whole-cell PCR as described previously (Read *et al.*, 2007).

To assay Gluc enzyme activity present in SCM, strains secreting the Gluc protein were grown in triplicate 20 mL YPGal cultures for 40 h at 30 °C with shaking. Gluc activity was measured by mixing 25 μL of SCM and 50 μL of 1 ×*Gaussia* luciferase assay buffer (New England Biolabs) in a Microfluor black flat-bottom microtiter plate (Thermo Labsystems, Franklin, MA). Luminescence was immediately measured in an LMax luminometer (Molecular Devices) in relative light units (RLU). To limit any effect that variation in culture density had on luciferase abundance, RLU were normalized to the cell density of each culture (OD_600 nm_ units).

### Human interferon Hy3 expression

For secreted expression of interferon Hy3 in *K. lactis*, the Gateway destination vector pDest-920 was created by Gateway conversion of the integrative *K. lactis* expression vector pKLAC1 (Colussi & Taron, 2005) as follows. Vector pKLAC1 was digested with XhoI (New England Biolabs) and the cohesive ends were filled in with Klenow DNA polymerase (New England Biolabs) to produce a blunt-ended DNA fragment that was ligated to the Gateway reading frame B cassette (Invitrogen, Carlsbad, CA). The ligation reaction was used to transform *Escherichia coli* DB3.1 cells. Colonies were selected on Luria–Bertani medium containing 100 μg ampicillin mL^−1^ and 15 μg chloramphenicol mL^−1^, and cloned vectors were screened by restriction digest for insert orientation. Correct clones were sequenced through the Gateway cassette junctions to ensure that the reading frame was maintained.

Interferon Hy3 Gateway entry clones were generated as described previously (Esposito *et al.*, 2005). These clones contained sequences for reconstitution of the Kex protease site (KR↓) and two Ste13p cleavage sites (EA↓EA↓) immediately upstream of the start codon of the interferon Hy3 gene (GenBank AF085805). Entry clones were recombined into the expression vector pDest-920 using Gateway LR recombination (Invitrogen) as per the manufacturer's protocols to generate pDest-920-IFN. Assembled pDest-920-IFN clones were verified by restriction digestion, and high-quality DNA was prepared using the GenElute XP Midiprep Kit (Sigma-Aldrich). Ten micrograms of pDest-920-IFN were digested with SacII (New England Biolabs) and the linear expression cassette was isolated, concentrated using a QiaQuick spin column (Qiagen, Valencia, CA) and used to transform each of the *K. lactis* protease null mutant strains.

### Fed-batch *K. lactis* fermentation

Yeast defined fermentation medium (YDFM) was composed of (per liter) 11.83 g KH_2_PO_4_, 2.29 g K_2_HPO_4_, 30 g glucose, 1 g MgSO_4_·7H_2_O, 10 g NH_4_SO_4_, 0.33 mg CaCl_2_·2H_2_O, 1 g NaCl, 1 g KCl, 5 mg CuSO_4_·5H_2_O, 30 mg MnSO_4_·H_2_O, 8 mg Na_2_MoO_4_·2H_2_O, 10 mg ZnCl_2_, 1 mg KI, 2 mg CoCl_2_·6H_2_O, 0.4 mg H_3_BO_3_, 30 mg FeCl_3_·6H_2_O, 0.8 mg biotin, 20 mg Ca-pantothenate, 15 mg thiamine, 16 mg *myo*-inositol, 10 mg nicotinic acid and 4 mg pyridoxine. The phosphate buffer and glucose were sterilized in the bioreactor after which the remaining components were added from sterile stock solutions after cooling.

A 1 L seed culture of the *K. lactis*Δ*yps1* background carrying an integrated vector for expression of interferon Hy3 was grown to mid-log phase (OD_600 nm_≍0.2–1.0 mL^−1^) in YPD medium, after which 100 mL was used to inoculate 1 L of YDFM in a Bioflow 110 fermentor (New Brunswick Scientific, Edison, NJ). The culture was grown for 18.75 h (OD_600 nm_≍40–50 mL^−1^) until a decline in the growth rate was detected using a BugEye 100C noninvasive biomass monitor (BugLab, Danville, CA) indicating a depletion of nutrients from the batch phase. At this point, a glucose feed was started. The glucose feed medium consisted of (per liter) 500 g glucose, 10 g MgSO_4_·7H_2_O, 16.5 g CaCl_2_·2H_2_O, 1 g NaCl and 1 g KCl. Trace metals and vitamins were added to 1.5 and four times the concentration present in YDFM, respectively. Glucose feeding was performed for 4 h at 0.38 mL min^−1^, after which the feed was stopped and a galactose feed was initiated to induce production of interferon Hy3. The galactose feed medium had the same composition as glucose feed medium, except 500 g galactose was substituted for glucose. Galactose feeding was performed for 24 h at 0.38 mL min^−1^ after which the culture was chilled to 10 °C for collection of SCM containing interferon Hy3. After chilling, the culture was centrifuged at 4000 ***g*** for 15 min to remove cells. The SCM was filtered by passage through a Sartopore 2 capsule (0.2 μM; Sartorius Stedim, Aubagne, France) and was immediately stored at 4 °C.

### Western blotting

Western blotting was used to assess the quality of the Gluc and interferon Hy3 proteins secreted from various *K. lactis* strains. SCM (10 μL) was analyzed by denaturing polyacrylamide gel electrophoresis (SDS-PAGE) on a 10–20% Tris-glycine polyacrylamide gel (Cosmo Bio Company, Tokyo, Japan). Separated proteins were transferred to nitrocellulose membrane (Whatman GmbH, Dassel, Germany). For detection of Gluc, the membrane was probed with an anti-GLuc antibody (1 : 3000 dilution; New England Biolabs), followed by a horseradish peroxidase (HRP)-conjugated anti-rabbit secondary antibody (1 : 2000 dilution; Cell Signaling Technology, Danvers, MA). For detection of interferon Hy3, the membrane was probed with a rabbit polyclonal antibody (1 : 1000 dilution) generated against the C-terminal tail of Hy3 (Covance, Princeton, NJ) followed by an HRP-conjugated anti-rabbit secondary antibody. Protein-antibody complexes were visualized using either LumiGlo™ (Cell Signaling Technology) or West Pico (ThermoFisher Scientific) detection reagents.

## Results and discussion

### Identification of pfam00026 aspartyl proteases encoded by *K. lactis*

The pfam00026 protein family is a highly conserved family of eukaryotic aspartyl proteases. We examined the distribution of putative pfam00026 proteins encoded in 12 sequenced yeast genomes from nine different genera (Table S2). There was wide variation in the total number of pfam00026 proteins encoded in these yeasts with as few as five (*K. lactis* and *Vanderwaltozyma polyspora*) to as many as 39 (*Yarrowia lipolytica*). The proteins identified were predominantly secretory proteins, with 91% having an obvious secretion peptide as predicted by signalp (Bendtsen *et al.*, 2004) and 21% also having a putative C-terminal glycosylphosphatidylinositol anchor attachment site as modeled by the Big-PI predictor algorithm (Eisenhaber *et al.*, 1999), suggesting that they are covalently associated with the plasma membrane or cell wall.

Analysis of the *K. lactis* genome identified five potential pfam00026 proteins, four being obvious counterparts to *S. cerevisiae* Yps1p, Yps7p, Bar1p and Pep4p as determined by blastp (Altschul *et al.*, 1997) analysis (Table 1). The fifth protein, encoded by locus KLLA0D01507g, showed lesser sequence similarity to ScYps6p (blastp*e*-value=3.6 × 10^−11^), suggesting that it might be a *K. lactis* yapsin family protease. Interestingly, *K. lactis* lacked obvious counterparts to the *S. cerevisiae* yapsin pfam00026 proteins Yps3p, Mkc7p and Yps5p. Finally, of the five putative *K. lactis* aspartyl proteases identified, three (Yps1p, Yps7p and KLLA0D01507p) have been detected in the culture medium of *K. lactis* GG799 cells propagated at high density in a bioreactor (Swaim *et al.*, 2008; Madinger *et al.*, 2009). Not knowing *a priori* as to which pfam00026 proteases may be most detrimental to heterologous proteins secreted from the *K. lactis* GG799 manufacturing strain, we elected to construct a set of five individual pfam00026 protease null mutants and assess each strain for its growth characteristics, protein expression performance and ability to improve recombinant protein quality.

**Table 1:** *Kluyveromyces lactis* putative pfam00026 aspartyl proteases

GenBank™accession	Locus tag	Protein length (a.a.)	SP cleavage site*	GPI omega site†	Closest *S. cerevisiae* homolog	blastp*e*-value‡
XP_454126	KLLA0E03938g	589	Ala-18	Gly-562	Yps1p	7.5 e-122
XP_456066	KLLA0F22088g	558	Ala-19	ND	Yps7p	8.8 e-80
XP_453761	KLLA0D15917g	511	Cys-18	Gly-490	Bar1p	1.2 e-84
XP_453326	KLLA0D05929g	409	Ala-25	ND	Pep4p	3.6 e-161
XP_453136	KLLA0D01507g	515	Ala-29	ND	Yps6p	3.6 e-11

* Putative signal peptide cleavage sites were predicted using signalp 3.0 (Bendtsen *et al.*, 2004).

† Putative glycosylphosphatidylinositol (GPI) anchor attachment (omega) sites were predicted using the big-pi predictor (Eisenhaber *et al.*, 1999).

‡ blastp searches were performed at the SGD website (http://yeastgenome.org). The presented *e*-values reflect homology to the closest *Saccharomyces cerevisiae* protein sequence.

ND, not defined.

### Construction of marker-free protease mutant strains

Seemingly innocuous alterations to a wild-type industrial expression strain may dramatically impact its expression performance. In one such example, introduction of a common uracil auxotrophy (Δ*ura3*) into the *K. lactis* GG799 background almost completely abolished heterologous protein expression, even when growth media were supplemented with exogenous uracil (or uridine) or when uracil prototrophy was restored by complementation *in trans* by expression of *URA3* (data not shown). A similar phenomenon has been reported for protein expression in certain *S. cerevisiae* strains carrying nutritional auxotrophies (Görgens *et al.*, 2004). Thus, we elected to perform genetic modifications in the *K. lactis* GG799 expression background without the use of auxotrophic genetic markers. Additionally, to preserve our ability to perform multiple genetic manipulations without requiring several different antibiotic resistance genes, we adopted the use of a dominant selectable marker recycling gene disruption strategy based on the *A. nidulans amdS* gene encoding acetamidase.

A method for using *amdS* to create selectable marker-free *K. lactis* strains was first described in a patent by Selten *et al.* (1999). This strategy relies on the unique ability of expressed acetamidase to be used in both positive selection and counterselection schemes. As a positive selection, cells transformed by a DNA construct containing *amdS* (e.g. an expression vector or gene disruption fragment) can grow on nitrogen-free medium containing acetamide. They can process the acetamide to ammonia, which is consumed by cells as the sole source of nitrogen (Selten *et al.*, 1999; Colussi & Taron, 2005; Read *et al.*, 2007). As a counterselection, cells transformed by an integrative *amdS*-containing vector produce acetamidase that processes fluoroacetamide provided in the growth medium to the toxic compound fluoroacetate. Thus, only cells that have been cured of the *amdS* gene through vector out-recombination are able to survive on plates containing fluoroacetamide.

In this study, we devised a PCR-based approach for rapid construction of DNA fragments for targeted deletion of genes using *amdS* for both transformant selection and subsequent marker recycling (Fig. 1). This method was used to create strains carrying marker-free null alleles of each of the five *K. lactis* pfam00026 aspartyl protease genes. For each protease gene, a disruption fragment was created by PCR amplification of the insert from vector pCT468 (see Materials and methods) while typically introducing 160–250 bp of DNA homologous to the target chromosomal locus on either end of the fragment (Fig. 1a). Each disruption fragment was separately introduced into *K. lactis* GG799 cells whereupon it integrated at the target locus through homologous recombination (Fig. 1b). *Kluyveromyces lactis* cells with disrupted target alleles were identified by genomic PCR (Fig. 1c and d) or Southern hybridization (not shown). Correct targeting of disruption fragments to the *yps1*, *yps7*, *pep4*, *bar1* and *KLLA0D01507g* loci occurred in 15%, 8%, 17%, 3% and 1% of transformants, respectively. The *amdS* marker was recycled from disrupted strains by growth on medium containing fluoroacetamide to counterselect for survival of cells that out-recombined the *amdS* marker through homologous recombination of the directly repeating flanking UTR regions (Fig. 1b). Out-recombination of the *amdS* marker leaves a 300-bp UTR ‘scar’ on the chromosome.

While this method was used to introduce a null mutation at a single locus in the present study, it can also be used iteratively to disrupt multiple chromosomal loci in the same background. At present, this PCR-based approach has been used to create *K. lactis* GG799 strains carrying five different null alleles through successive rounds of gene deletion and *amdS* marker-recycling (M. Ganatra & C. Taron, unpublished data). Additionally, it is conceivable that this PCR-based method could be extended for use with other yeast species when deletion of chromosomal regions in prototrophic strain backgrounds is desired.

### Growth characteristics of protease deletion mutants

The overall health of a genetically modified yeast expression strain can significantly impact the yield of both secreted and intracellular heterologous proteins. We therefore investigated whether each of the aspartyl protease mutant strains exhibited health or growth defects.

**Fig. 2:**
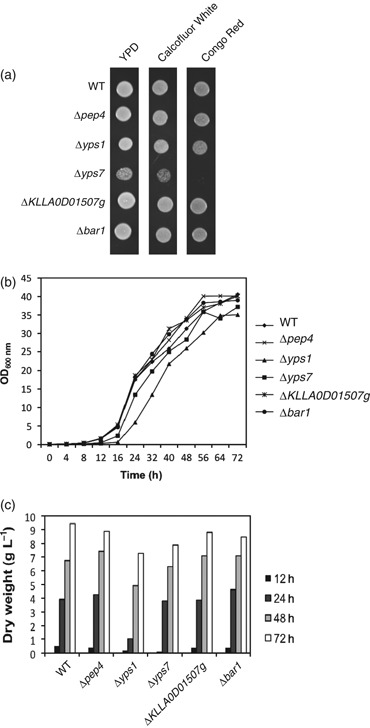
Growth characteristics of *Kluyveromyces lactis* protease deletion mutant strains. (a) Protease deletion mutants were propagated in the presence of the cell wall-perturbing dyes Calcofluor White (10 μg mL^−1^) and Congo Red (200 μg mL^−1^). Spots represent cell growth after 3 μL of a cell suspension (0.2 OD_600 nm_ units) was placed onto YPD agar (with or without dye) and incubated at 30°C for 3 days. (b) Growth of wild-type (wt) cells and each protease mutant over 72 h of culturing in YPGal medium. (c) Dry biomass produced by each mutant strain grown in liquid YPGal medium after 12, 24, 48 and 72 h.

Strains were first examined for obvious cell morphology phenotypes using phase-contrast microscopy. No aberrant cellular morphologies were observed for the Δ*yps1*, Δ*pep4*, Δ*bar1* and Δ*KLLA0D01507g* strains grown in YPGal medium. However, Δ*yps7* formed small aggregates of cells (data not shown), suggesting that this mutant may have a defect in maintenance of the cell wall. Prior studies have shown that the yapsin aspartyl protease family is important for maintaining cell wall integrity in *S. cerevisiae* and *C. glabrata* (Krysan *et al.*, 2005; Kaur *et al.*, 2007). Therefore, the integrity of the cell wall of each of the null mutants was tested by assessing their growth in the presence of the cell wall-disrupting compounds Calcofluor White and Congo Red (Kopecká & Gabriel, 1992; Ram *et al.*, 1994). The Δ*yps7* mutant displayed complete sensitivity to 200 μg mL^−1^ Congo Red and partial sensitivity to 10 μg mL^−1^ Calcofluor White (Fig. 2a), indicating that it has a weakened cell wall, further suggesting that Yps7p plays a role in the maintenance of cell wall integrity in *K. lactis*. The same growth phenotype was described previously for Δ*yps7* mutants in both *S. cerevisiae* (Krysan *et al.*, 2005) and *C. glabrata* (Kaur *et al.*, 2007). However, in *S. cerevisiae*, Δ*yps1* cells showed complete sensitivity to 200 μg mL^−1^ Congo Red (Krysan *et al.*, 2005), whereas the same concentration had little effect on *K. lactis*Δ*yps1* cells (Fig. 2a), suggesting that this mutation may be less severe in *K. lactis*.

The ability of the protease deletion mutants to grow effectively in liquid culture was assessed by analysis of growth kinetics and measurement of dry cell biomass produced over 72 h (Fig. 2b and c). The wilt-type (wt), Δ*pep4*, Δ*bar1* and *KLLA0D01507g* strains had a similar lag phase (∼12 h), doubling time in log phase growth (2.8 h, calculated after 24 h) and density at saturation (∼40 OD_600 nm_ units and ∼9 g L^−1^ dry mass). The Δ*yps7* strain had a similar lag phase (∼12 h), but grew slightly slower (3.0 h doubling time), and reached a slightly lower cell density at saturation (∼38 OD_600 nm_ units and ∼8.2 g L^−1^ dry mass). The Δ*yps1* mutant had a significantly longer lag phase (∼18 h) and grew slower in log phase (3.5 h doubling time) than the other strains. However, after 72 h, this strain ultimately reached a saturation point (∼36 OD_600 nm_ units and ∼7.5 g L^−1^ dry mass) that was ∼90% that of wild-type cells. One possible explanation for the longer lag phase of *K. lactis*Δ*yps1* may be that these cells lose viability in stationary phase. This phenomenon was shown previously for the *C. glabrata*Δ*yps1* strain (Kaur *et al.*, 2007).

### Protease activity secreted by the deletion mutants

Two approaches were used to assess total secreted protease activity directly in SCM derived from cultures of each mutant. These methods involved testing the stability of fluorescently labeled casein and measuring the hydrolysis of a chromogenic peptide substrate containing dibasic amino acids. Additionally, growth of yeast cells on different carbon sources may either increase or repress the expression of individual proteases and influence the proteins that cells secrete (Sinha *et al.*, 2004; Madinger *et al.*, 2009). This concept is of particular importance for *K. lactis* because of the common use of the carbon catabolite-controlled *LAC4* promoter to drive expression of recombinant genes (Colussi & Taron, 2005; van Ooyen *et al.*, 2006). Therefore, we compared protease activity in SCM from each strain propagated in glucose (YPGlu)-, galactose (YPGal)- or glycerol (YPGly)-containing medium.

**Fig. 3:**
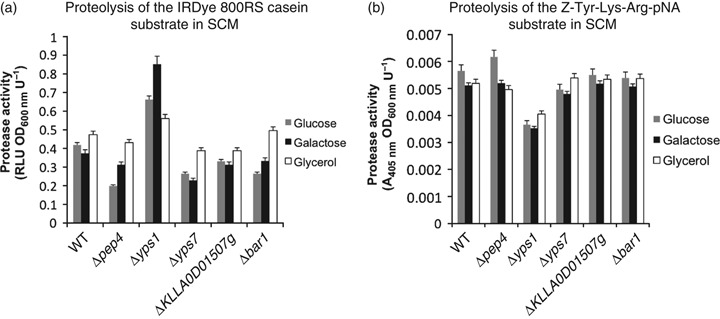
Total extracellular protease activity in spent culture medium (SCM) of protease mutant strains. Protease activity in SCM of the individual strains assayed using (a) IRDye casein or (b) the peptide Z-Tyr-Lys-Arg-pNA as a substrate.

As an assay for general protease activity present in SCM samples, hydrolysis of the fluorescent protease substrate IRDye 800RS-labeled casein (IRDye-casein) was measured (Fig. 3a). When propagated in YPGlu or YPGal medium, SCM from Δ*yps7*, Δ*pep4*, Δ*KLLA0D01507g* and Δ*bar1* strains hydrolyzed IRDye-casein less than SCM from wt cells (Fig. 3a, gray and black bars). This was most pronounced for the Δ*yps7* and Δ*pep4* strains. SCM from the same four strains each also showed higher levels of activity for cells grown in YPGly medium (Fig. 3a, open bars) compared with YPGlu or YPGal. The most notable difference was a significant increase in general protease activity in SCM of the Δ*yps1* mutant grown on each carbon source. This increase was most pronounced in SCM from cells propagated in YPGal (>2-fold). It is possible that in the absence of Yps1p other secretory proteases become more abundant, suggesting that Yps1p may modulate the activity of other secretory proteases or lack of Yps1p induces a stress response that results in increased production of other proteases.

Yapsin family proteases have a preference for cleavage of proteins at basic amino acids. Thus, to generally assess the protease activity in SCM attributable to yapsin family proteases, an internally quenched chromogenic peptide substrate, Z-Tyr-Lys-Arg-pNA, was used. This substrate was used previously to assay the activity of human furin, a subtilisin-like processing protease (Rozan *et al.*, 2004). However, the Lys–Arg motif within this substrate is also a well-characterized cleavage site for yapsin proteases (Komano *et al.*, 1999; Olsen *et al.*, 1999). Cleavage of this peptide substrate in SCM derived from the Δ*yps1* mutant grown in YPGlu, YPGal and YPGly was reduced by 34%, 32% and 22% compared with corresponding SCM from wt cells, respectively (Fig. 3b). In contrast, peptide stability in SCM of the Δ*yps7* yapsin mutant and other protease null mutants was comparable to the wt strain. These data support the conclusion that Yps1p contributes significantly to proteolysis at dibasic residues in *K. lactis*.

### Heterologous protein expression in protease null mutant strains

The individual protease null mutants were tested for their ability to efficiently express heterologous proteins and to improve the quality of secreted proteins. Two proteins that experience partial proteolysis when secreted from wild-type *K. lactis* GG799 cells were examined: a naturally secreted luciferase from the copepod *G. princeps* (Verhaegent & Christopoulos, 2002) and a human chimeric interferon with antiviral properties (Hu *et al.*, 1999).

**Fig. 4:**
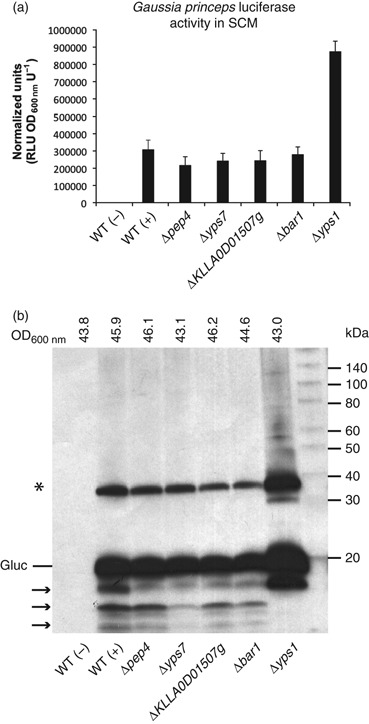
Secretion of *Gaussia princeps* luciferase by protease-deficient strains. (a) Secreted *Gaussia* luciferase (Gluc) enzyme activity present in spent culture medium (SCM) derived from strain GG799 (wt) (± an expression vector) and each protease mutant was measured by luciferase assay. (b) Western blot analysis of recombinant Gluc in SCM. Arrows indicate Gluc proteolysis products and the asterisk indicates dimerized Gluc. The final cell density (OD_600 nm_ units) of each culture is indicated above each lane.

*Gaussia princeps* luciferase (Gluc) was expressed in wt GG799 cells, and each of the protease null mutants and secreted Gluc activity was assayed directly from SCM of strains cultured in YPGal medium. Comparable levels of Gluc activity were secreted from Δ*pep4*, Δ*yps7*, Δ*KLLA0D01507g*, Δ*bar1* and wt cells, whereas more than threefold more Gluc activity was secreted from Δ*yps1* (Fig. 4a). Western blotting with an anti-Gluc antibody was used to detect the ∼20-kDa Gluc protein and to qualitatively assess its susceptibility to proteolysis (Fig. 4b). Gluc secreted from wt cells and the Δ*pep4*, Δ*yps7*, Δ*KLLA0D01507g* and Δ*bar1* mutants experienced similar levels of proteolysis (Fig. 4b, arrows). However, Gluc proteolysis was significantly reduced in the Δ*yps1* strain background, correlating with the large increase in Gluc activity observed for this strain.

**Fig. 5:**
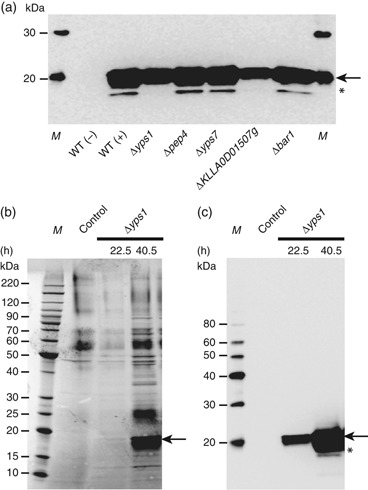
Secretion of human interferon Hy3 by protease-deficient strains. (a) Western blot analysis of recombinant Hy3 present in SCM derived from strain GG799 (wt) (± an expression vector) and each protease mutant. (b, c) Analysis of Hy3 secreted by the *Kluyveromyces lactis*Δ*yps1* mutant grown to high cell density in a bioreactor. Hy3 secreted after 22.5 and 40.5 h of fermentation was visualized by SDS-PAGE separation of SCM and either Coomassie blue staining (b) or Western blotting (c). SCM from GG799 cells not expressing Hy3 was analyzed as a negative control. In all panels, arrows indicate intact Hy3 protein and M represents a molecular weight marker. In (a) and (c), the asterisk denotes a proteolytic product of Hy3.

In a similar experiment, the chimeric human interferon Hy3 was expressed in wt GG799 cells and each of the protease null mutants. Western blotting with an anti-Hy3 antibody was used to visualize the ∼20-kDa Hy3 protein and the products of its proteolysis in SCM from cultures grown in shake flasks. Significant proteolysis of secreted Hy3 was observed for all strains except Δ*yps1* and Δ*KLLA0D01507g* where detrimental processing was nearly abolished; however, the level of Hy3 expression produced in the Δ*KLLA0D01507g* background was slightly reduced compared with Δ*yps1* cells. The Δ*yps1*-Hy3 expression strain was also propagated to high cell density (OD_600 nm_=187) in a 1-L bioreactor using a fed-batch fermentation strategy to simulate a typical manufacturing process. Under these conditions, proteolysis of Hy3 was not observed (Fig. 5b). In a highly overexposed Western, a faint band probably represents a trace amount of proteolytic product (Fig. 5c, 40.5 h, asterisk), but comparison with the Western analysis of Hy3 expressed in the wild-type strain (Fig. 5a) shows that the Δ*yps1* mutation has significantly improved the quality of this protein. In addition, the yield of Hy3 in this unoptimized fermentation was estimated at ∼300 mg L^−1^ (data not shown), suggesting that the Δyps1 mutant strain is a viable host for further development of larger-scale production processes.

## Conclusions

We identified five pfam00026 apartyl protease genes encoded by the *K. lactis* genome and developed a PCR-based strategy to construct a set of selectable marker-free deletion strains in the *K. lactis* GG799 manufacturing strain background. Each mutant strain was altered in its levels of secreted protease activity compared with wt cells, and each functioned efficiently as a host for heterologous protein expression. In particular, the Δ*yps1* mutant was a compelling strain for improving heterologous protein expression despite having a longer lag phase and slower growth than the other strains. This strain background showed significant improvement in the quality and yield of two proteolysis-prone proteins in shake-flasks and at high cell density in a bioreactor. This set of *K. lactis* mutants represents an important tool for improving expression of protease-sensitive proteins in the GG799 strain background.

## Statement

Re-use of this article is permitted in accordance with the Terms and Conditions set out at: http://wileyonlinelibrary.com/onlineopen#OnlineOpen_Terms

## Supporting information

**Table S1.** Oligonucleotide primers for PCR.

**Table S2.** Yeast putative pfam00026 aspartyl proteases.
