# Supplementary material for: A set of aspartyl protease-deficient strains for improved expression of heterologous proteins in Kluyveromyces lactis
Source: FEMS Yeast Res. 2010 Dec 17;11(2):168–78. doi: 10.1111/j.1567-1364.2010.00703.x (PMC3041862; doi:10.1111/j.1567-1364.2010.00703.x)
Supplement: Supplementary file 1 [file fyr0011-0168-SD1.pdf]

Supplemental Information

A set of aspartyl protease-deficient strains for improved expression of heterologous proteins in *Kluyveromyces lactis*

Mehul B. Ganatra, Saulius Vainauskas, Julia M. Hong, Troy E. Taylor, John-Paul M. Denson, Dominic Esposito, Jeremiah D. Read, Hana Schmeisser, Kathryn C. Zoon, James L. Hartley and Christopher H. Taron*

*Correspondence to: Christopher H. Taron, New England Biolabs, 240 County Road, Ipswich, MA 01938-2723, USA
E-mail: taron@neb.com
Telephone: 978-927-5054, Fax: 978-921-1350

Table of Contents

Table S1. Oligonucleotide primers for PCR.

A table disclosing all oligonucleotide primers used in construction of gene deletion fragments by PCR. Primers used for genotyping mutant strains by genomic PCR are also provided.

Table S2. Yeast putative pfam00026 aspartyl proteases

A table listing all pfam00026 aspartyl proteases encoded by currently sequenced yeast genomes.

Table S1. Oligonucleotide primers used for PCR.

Gene Name	Primer Name[a]	Nucleotide Sequence (5' to 3')[b]
PEP4	KO1	**GGTCAGATATATAGTAGTGTCAGTATTTTGAACGGAGAGG**<br>**AACTTGATTAAATCTATTATACAGTTTCCCCCAAAATTTTTC**<br>**TGAAATTGTGCCGCTAACTGTTCATTAAACGGTGCTTTCTT**<br>**ACAACAAAAAAATAAGC**GATCCCCTCGCGAGTTGG
	KO2	CCGTCTCCTGACCCTCCAT
	KO3	GGCGTCATCGGTGTAGGA
	KO4	**CTAATTCGATTTGTGAAACTGACATTCAATTAATATAGCAG**<br>**TAAGTTAACCGTCTTATTTAATTATTTTTTTAGAGTTGTCGA**<br>**GTTAATAGAAGCTGAACTCCAATTCAAAAGGTTACAATTC**<br>**GATCGCTTTTTTAACAA**TAATGCATCAAGAAACTGTCG
	KO5	**TATCAAATATAAATACGGTAAAAAGAAGGGGCGATTAATT**<br>**TTGCCGTTTATGCAATAACCTTGTTTTAAGGGTATCAATGT**<br>**CTGAATAATGTCATCGATAATAGAAGTGTTGACATAACTAT**<br>**ATTAAAGACAGGGTAGAC**GGTCAGATATATAGTAGTGT
	KO6	**CAAAGCCAAAGCCAATTGAATAATGTCCATCGTCTATATAT**<br>**TTAGAGGAAGGAACACAATTGTTTTATGATGAAATAGGAA**<br>**AGAATTCTTTTAGGTATTTTAAGGTTTATTTGTCACCAAGT**<br>**GTTGTTATACTTTGTTAG**CTAATTCGATTTGTGAAACT
	ID1	GGGTGCACACAAATTGCACT
	ID2	GGCAATTTATCGACATTTTA
	ID3	AGAAAGATTCTTGAGGGTAAG
	ID4	ATTACTTCAATAGATGGCAA
YPS1	KO1	**TAAAGGGTCTTTTCATTTTACAGAAGACGTTATACTTTGGC**<br>**TGACGTCTTTTTTTCTTTAATAATTAAAGTTCATTTCTTGGT**<br>**ACTGGTTACTAATTCCCCCTTTTATTATCCAAGGCGCATAT**<br>**ATAGCAAACACCAGCA**GATCCCCTCGCGAGTTGG
	KO2	CCGTCTCCTGACCCTCCAT
	KO3	GGCGTCATCGGTGTAGGA
	KO4	**ATGAAATAGACGAAGAGAGTAAGACCAAATGTGAGATGA**<br>**TGTGAAACGAAGTTCCTCTGATAGAAGTCTGCATCGTGT**<br>**GTGTTTGTGTGTATTTTTCTTTTTTTTTCTTTTGCTTTTATTA**<br>**AGTAACAGTCTTCCTCAGC**TAATGCATCAAGAAACTGTCG
	KO5	**TTCCCACGATTAAACGTACACTGGAAAATGTTTCTACCCA**<br>**TCTTGCATTTCACATCACGTACCTTCTCTCCTAAAAGTTTG**<br>**AGGTAACTGACTCATTGAATTGGAAAGAAAACAGCGGCT**<br>**ATTGTCTTAGCGTATATATA**TAAAGGGTCTTTTCATTTTA
	KO6	**ATTCTGATTATAGGCTTTCTGTTCCTATGCAAGTGGAACG**<br>**GAGATCGAGCAACGCATCAATTTTTGGGCAGTGAAAGGG**<br>**TTTCTATTGGCAATAGAATCTTTGATCTGACAAAGAACCG**<br>**AAAATGTGCTAAATTAAGACA**ATGAAATAGACGAAGAGAG
	ID1	GGAACTTTGAAACGTTGAAA
	ID2	GAAATTGAATTAACTAGGGT
	ID3	AACCATCTGCACGCTTCTTCAAAT
	ID4	ATTACTTCAATAGATGGCAA
YPS7	KO1	**TCTGTTGGTCTAATTCTTTCCTTTTGGATTACACACCATAA**<br>**AAGCATAGAGGAAGCGCGGTTTGTTCAACAATACGTTCG**<br>**CTATATTCTGGTTATTTTTCTTTGATCTGGATAATTGATATAT**<br>**ACAACGGACCTGCACAA**GATCCCCTCGCGAGTTGGTTC
	KO2	CCGTCTCCTGACCCTCCAT
	KO3	GGCGTCATCGGTGTAGGA

	KO4	**CAAACTAATGGTTAGACTGAATGATAGGTGATAATTGGAATGTTCTACATCTCTATAGTTAATCCTTGCTGTGAAGTCTTTATCTCCTGCTTATTTTGATTTTTCGTTAGATGTCGAAAAAAAGTTGTCAGAACTTGAGA**TAATGCATCAAGAAACTGTCG
	KO5	**TGAAGCTACAAACGAAAATAAGCAGAGCCTCGGGTGTGCAGCATATATCAACAGATACCGTGTATCCTCAGGCGCAACACCTACCATTTCGTGTGTGTTAAAAAGGTGTTTATCCTTTTATATCTGTGCTTTTTCTTTAC**TCTGTTGGTCTAATTCTTTC
	KO6	**AACATTATAAACTGCAAATGATTTTTAGGGTTCGATTCGGAGATGTCAGTTGATCTTAAACGACGTTGAACAACTTTGACCTTGGTTAATTGAAAGTCATTAATCAGGAGTTAAAATGATAAGGAATCTGAAAACATATG**CAAACTAATGGTTAGACTGA
	ID1	AGAAAACTGAAAATGTATAAA
	ID2	TGCTGCGACAAGTACGTATT
	ID3	AGGATAAATTTCACCGGACA
	ID4	ATTACTTCAATAGATGGCAA
KLLA0D01507g	KO1	**CCTTTGTTTTTATTATATTGGTGCTGTTTTTTTTTGCTTTCTCCTACGCATATTTTCTCCTCTGTCTCTTGTTTGAGATC**GATCCCCTCGCGAGTTGG
	KO2	CCGTCTCCTGACCCTCCAT
	KO3	GGCGTCATCGGTGTAGGA
	KO4	**ATTCATTGAAAATGTGAGAGGGCACTTCAGTAAAAGAGAATAAAAAGTGCTATTTTTACTCAGTAGTGGGATAATTTATT**TAATGCATCAAGAAACTGTCG
	KO5	**TCCAAAGAAAGTGATAACTTTTCTTTGCTTCTTGCTTGATAGATTCTGGTTCAATTGAGT**CCTTTGTTTTTATTATATTGGTG
	KO6	**TTGATTATAATGTACAATGATGGGGTTTTATCGTTCGGAGTTGCATGGACTGCGTCTATA**ATTCATTGAAAATGTGAGAG
	ID1	TTGATATTTTGGTCTGGTAGA
	ID2	AGAGTCATCTTCAATCCTATT
	ID3	TTGTTTACTTAATGCATCAT
	ID4	ATTACTTCAATAGATGGCAA
BAR1	KO1	**GCTCAGATAGTTGAATGATATCGTCATCTTCTTATTCCCAATTCATATTACATAGTTGGTAGGTATCAACGAAATACACC**GATCCCCTCGCGAGTTGG
	KO2	CCGTCTCCTGACCCTCCAT
	KO3	GGCGTCATCGGTGTAGGA
	KO4	**GCTCTTAAGCCTTAACGTAGGCAAACGGTCGTTTGTGCTATTGCGTCAGTTTTTTTCACCATTATCCAGCACTGACCGGAGAAGG**TAATGCATCAAGAAACTGTCG
	KO5	**GGCAATTACACAGTGACATCTTCAAGACGGCCATTAATAAAGTAAAACATATATAAAAGTACTAATTG**GCTCAGATAGTTGAATGATATCG
	KO6	**CGTTACCATGAAGCTAAACTGTGGGAATAAAGGTTCAGATAAGCAGAGTTTGATCCGTGATATTCATGCAAGAA**GCTCTTAAGCCTTAACGTAGG
	ID1	GTGGAAGGTTATTCGCAAAATAGTTC
	ID2	CCATTGAGTGTCAGTGATTC
	ID3	ATGGTGTACCTACACCAAGTTCA
	ID4	ATTACTTCAATAGATGGCAA

[a]Primers names correspond to those displayed in Fig. 1 of the main text.

[b]For primers KO1, KO4, KO5 and KO6, sequences in bold text represent the primer's "tail" that is homologous to the target chromosomal locus (see Fig. 1 in the main text).

Table S2. Yeast putative pfam00026 aspartyl proteases.

GenBank™ Accession	Locus tag or gene	Protein length (a.a.)	SP cleavage site[a]	GPI omega site[b]	Closest *S. cerevisiae* protein	Blastp e-value[c]
Ashbya gossypii						
NP_986475	AGOS_AGL192W	499	Cys-18	Asn-472	Mkc7p	1.3 e-97
NP_986906	AGOS_AGR240W	452	Ala-21	n.d.	Bar1p	7.0 e-71
NP_983552	AGOS_ACR150W	553	Ala-28	Gly-527	Yps7p	5.5 e-55
NP_983546	AGOS_ACR144W	408	Ala-20	n.d.	Pep4p	1.6 e-94
NP_983545	AGOS_ACR143W	393	Ala-18	n.d.	Pep4p	1.3 e-48
NP_982824	AGOS_ABL123C	494	Cys-17	n.d.	Pep4p	2.6 e-12
NP_987073	AGOS_AGR407C	268	n.d.	n.d.	Pep4p	3.4 e-49
Candida albicans						
XP_712692	*SAP9*	544	Ala-17	Ala-521	Yps1p	2.3 e-69
XP_717243	*SAP10*	453	Cys-20	Cys-433	Yps3p	2.6 e-31
XP_719105	*SAP6*	418	Ala-18	n.d.	Yps1p	2.2 e-45
XP_717988	*SAP4*	417	Ala-18	n.d.	Yps3p	2.2 e-42
XP_719941	*SAP8*	405	Ala-25	n.d.	Yps1p	7.6 e-40
XP_713566	*SAP7*	588	Ala-16	n.d.	Yps1p	1.9 e-38
XP_719147	*SAP5*	418	Ala-18	n.d.	Yps1p	7.6 e-47
XP_723063	*SAP3*	398	Ala-18	n.d.	Yps1p	7.6 e-49
XP_711047	*SAP2*	398	Ala-18	n.d.	Yps3p	2.4 e-45
EAK99043	*SAP1*	391	Ala-21	n.d.	Yps3p	2.2 e-44
XP_713148	*APR1*	419	Ala-22	n.d.	Pep4p	7.8 e-143
XP_720977	*SAP99*	363	Ala-17	n.d.	Yps3p	1.9 e-10
XP_721098	*SAP98*	364	Ala-17	n.d.	Pep4p	2.6 e-8
XP_719529	CaO19.2082	435	Ala-16	n.d.	Yps1p	8.0 e-12
Candida glabrata						
XP_445768	CAGL0E01815g	519	Ala-15	Asn-497	Yps3p	3.5 e-87
XP_445767	CAGL0E01793g	516	Ala-15	Asn-492	Yps1p	1.9 e-84
XP_445770	CAGL0E01859g	505	Ala-13	Asn-484	Yps3p	3.2 e-80
XP_445765	CAGL0E01749g	482	Ala-15	n.d.	Mkc7p	3.1 e-84
XP_445769	CAGL0E01837g	521	Ala-16	Ser-498	Mkc7p	3.5 e-83
XP_445766	CAGL0E01771g	519	Ala-15	Asn-495	Mkc7p	5.1 e-84
XP_449529	CAGL0M04191g	601	Ala-18	Asp-580	Yps1p	4.4 e-142
XP_445764	CAGL0E01727g	539	Leu-16	Asn-519	Yps1p	9.6 e-77
XP_445750	CAGL0E01419g	519	n.d.	n.d.	Yps1p	2.1 e-85
XP_445771	CAGL0E01881g	508	n.d.	n.d.	Yps1p	1.8 e-73
XP_447804	CAGL0J02288g	541	Cys-19	n.d.	Bar1p	3.0 e-79
XP_449442	CAGL0M02211g	415	Ala-22	n.d.	Pep4p	3.0 e-125
XP_444870	CAGL0A02431g	587	Ala-18	n.d.	Yps7p	6.7 e-82
Debaryomyces hansenii						
XP_456845	DEHA0A12309g	566	Gly-18	n.d.	Yps1p	5.0 e-88
XP_458472	DEHA0C19206g	408	n.d.	Ser-381	Yps1p	4.0 e-59
XP_456485	DEHA0A03729g	429	Ala-22	n.d.	Mkc7p	1.3 e-58
XP_458750	DEHA0D07370g	492	Ser-25	n.d.	Yps1p	2.2 e-35
XP_460063	DEHA0E18326g	605	n.d.	n.d.	Yps7p	1.2 e-31
XP_458031	DEHA0C08921g	416	Ala-23	n.d.	Pep4p	1.0 e-147
XP_456622	DEHA0A06941g	418	Ala-38	n.d.	Yps3p	2.9 e-23
XP_458471	DEHA0C19184g	276	Gly-18	n.d.	Pry1p	5.7 e-37
XP_458473	DEHA0C19217g	98	Ser-20	n.d.	Mkc7p	7.1 e-08
Kluyveromyces lactis						
XP_454126	KLLA0E03938g	589	Ala-18	Gly-562	Yps1p	7.5 e-122
XP_456066	KLLA0F22088g	558	Ala-19	n.d.	Yps7p	8.8 e-80
XP_453761	KLLA0D15917g	511	Cys-18	Gly-490	Bar1p	1.2 e-84
XP_453326	KLLA0D05929g	409	Ala-25	n.d.	Pep4p	3.6 e-161
XP_453136	KLLA0D01507g	515	Ala-29	n.d.	Yps6p	3.6 e-11

Lodderomyces elongisporus						
XP_001523133	LELG_05679	485	Ala-22	n.d.	Mkc7p	5.5 e-21
XP_001527279	LELG_02108	652	Ala-22	Ser-633	Yps1p	1.7 e-77
XP_001527254	LELG_02083	406	Gly-23	n.d.	Yps1p	4.2 e-41
XP_001527275	LELG_02104	402	Gly-23	n.d.	Yps3p	6.2 e-47
XP_001527016	LELG_01845	429	Ala-26	n.d.	Pep4p	4.7 e-147
XP_001523682	LELG_05098	753	Ala-25	n.d.	Yps7p	4.8 e-24
XP_001523572	LELG_05418	394	Ala-30	n.d.	Yps6p	2.3 e-26
XP_001528529	LELG_01049	354	Ala-20	n.d.	Yps3p	3.3 e-16
Pichia guilliermondii						
XP_001486254	PGUG_01925	549	Ala-21	Ser-519	Yps1p	1.9 e-75
XP_001486625	PGUG_00002	504	Ala-17	n.d.	Mkc7p	4.1 e-66
XP_001482927	PGUG_04882	582	Ala-16	n.d.	Yps7p	4.6 e-36
XP_001483230	PGUG_03959	388	Ser-18	n.d.	Yps3p	1.1 e-38
XP_001483229	PGUG_03958	402	Ala-18	n.d.	Yps3p	2.2 e-44
XP_001486547	PGUG_02218	389	Ala-22	n.d.	Yps1p	7.9 e-33
XP_001483228	PGUG_03957	393	Ala-23	n.d.	Bar1p	3.2 e-38
XP_001482648	PGUG_05668	390	Ala-22	n.d.	Yps3p	2.0 e-31
XP_001483889	PGUG_03270	384	Ala-17	n.d.	Yps6p	1.0 e-23
XP_001484971	PGUG_02700	399	Ala-23	n.d.	Yps3p	1.6 e-39
XP_001484972	PGUG_02701	414	Gly-23	n.d.	Yps3p	2.7 e-37
XP_001483530	PGUG_04259	322	Ala-17	n.d.	Yps1p	3.0 e-40
XP_001483416	PGUG_04145	408	Ala-21	n.d.	Pep4p	5.9 e-145
XP_001486562	PGUG_02233	171	n.d.	n.d.	Bar1p	6.6 e-04
Pichia pastoris						
XP_002494022	PAS_chr4_0584	599	Ala-23	Gly-574	Yps1p	1.3 e-95
XP_002492372	PAS_chr3_1157	562	Ala-16	Ala-543	Yps1p	5.0 e-45
XP_002490038	PAS_chr1-1_0379	593	Ala-18	Asn-572	Mkc7p	4.7 e-50
XP_002492519	PAS_chr3_0299	527	Ala-18	Ser-502	Yps1p	5.8 e-44
XP_002492523	PAS_chr3_0303	473	Ala-22	n.d.	Yps1p	4.5 e-39
XP_002493099	PAS_chr3_0866	612	Ala-16	n.d.	Mkc7p	1.9 e-30
XP_002493333	PAS_chr3_1087	410	Ala-24	n.d.	Pep4p	1.5 e-157
XP_002492620	PAS_chr3_0394	582	Ala-16	Asn-555	Yps7p	1.8 e-23
Pichia stipitis						
XP_001386659	PICST_68459	570	Ala-21	n.d.	Yps1p	5.8 e-83
XP_001382514	PICST_29930	575	n.d.	n.d.	Yps7p	2.3 e-28
XP_001386187	PICST_63754	412	Ala-26	n.d.	Yps1p	7.7 e-59
XP_001387880	PICST_39213	335	n.d.	n.d.	Yps3p	1.0 e-44
XP_001385957	PICST_62133	431	Ala-18	n.d.	Yps3p	4.7 e-55
XP_001382965	PICST_40413	415	Ala-18	n.d.	Yps3p	4.8 e-37
XP_001386378	PICST_68393	572	Ala-17	n.d.	Bar1p	2.2 e-10
XP_001385673	PICST_73446	417	Ala-22	n.d.	Pep4p	5.9 e-145
XP_001382254	PICST_29382	450	Gly-16	n.d.	Yps6p	2.1 e-13
Saccharomyces cerevisiae						
NP_013221	*YPS1*	569	Gly-21	Ser-542	n.d.	n.d.
NP_010428	*MKC7*	596	Ala-22	Asn-575	n.d.	n.d.
NP_013222	*YPS3*	508	Gly-20	Asn-483	n.d.	n.d.
NP_012305	*YPS6*	537	Ala-24	Asn-515	n.d.	n.d.
NP_012249	*BAR1*	587	Ala-24	n.d.	n.d.	n.d.
NP_015171	*PEP4*	405	Ala-22	n.d.	n.d.	n.d.
NP_010636	*YPS7*	596	Ala-25	Asp-573	n.d.	n.d.
NP_076890	YGL258w	77	n.d.	n.d.	n.d.	n.d.
NP_011255	*YPS5*	165	Ala-24	n.d.	n.d.	n.d.
Vanderwaltozyma polyspora						
XP_001646089	Kpol_543p61	588	Ser-26	Gly-560	Yps3p	4.1 e-114
XP_001645003	Kpol_1072p15	399	Ala-18	n.d.	Pep4p	1.4 e-152
XP_001646193	Kpol_1013p6	402	Gly-21	n.d.	Pep4p	7.6 e-161

XP_001643269	Kpol_1063p22	336	Ser-24	n.d.	Bar1p	2.5 e-59
XP_001644201	Kpol_1059p33	662	Gly-24	n.d.	Yps7p	2.8 e-83
Yarrowia lipolytica						
XP_503768	YALI0E10175g	534	Ala-17	n.d.	Yps3p	1.1 e-72
XP_502815	YALI0D14300g	384	Ala-17	n.d.	Yps1p	2.3 e-37
XP_502045	YALI0C20273g	385	Ala-17	n.d.	Yps1p	1.4 e-42
XP_504204	YALI0E20823g	462	n.d.	n.d.	Yps1p	4.2 e-54
XP_499671	YALI0A02002g	443	Ala-22	n.d.	Yps3p	7.4 e-44
XP_500335	YALI0B00132g	476	Ala-17	n.d.	Yps1p	2.3 e-51
XP_501140	YALI0B20526g	392	Ala-20	n.d.	Yps3p	1.6 e-46
XP_500342	YALI0B00374g	389	Ala-18	n.d.	Bar1p	9.0 e-41
XP_501603	YALI0C08547g	385	Ala-17	n.d.	Yps3p	6.8 e-41
XP_501847	YALI0C14938g	447	Ala-17	n.d.	Yps1p	2.3 e-55
XP_501124	YALI0B20174g	393	Ala-17	n.d.	Yps1p	5.3 e-45
XP_504397	YALI0E25784g	393	Ala-17	n.d.	Yps3p	1.5 e-36
XP_502281	YALI0D01331g	457	Ala-17	n.d.	Yps1p	7.4 e-49
XP_500144	YALI0A16819g	457	Ser-18	Ser-435	Mkc7p	2.7 e-45
XP_504725	YALI0E33363g	397	Ala-17	n.d.	Mkc7p	6.6 e-36
XP_501434	YALI0C04279g	382	Ala-14	n.d.	Yps3p	8.8 e-25
XP_505251	YALI0F10549g	450	Ala-17	n.d.	Yps1p	4.1 e-46
XP_504265	YALI0E22374g	727	Ala-15	Ala-707	Yps3p	1.4 e-47
XP_501619	YALI0C08899g	359	Ala-18	n.d.	Bar1p	1.2 e-35
XP_503171	YALI0D22957g	455	Ala-17	n.d.	Yps1p	1.1 e-17
XP_501781	YALI0C12980g	430	Ala-44	n.d.	Pep4p	9.1 e-14
XP_502672	YALI0D10835g	778	Gly-18	n.d.	Yps3p	2.1 e-32
XP_501700	YALI0C10923g	416	Ala-14	n.d.	Yps1p	2.6 e-20
XP_503918	YALI0E13860g	378	Ala-20	n.d.	Yps3p	1.8 e-40
XP_501592	YALI0C08283g	374	Ala-15	n.d.	Pep4p	3.4 e-30
XP_504768	YALI0E34331g	597	Ala-15	Gly-569	Yps7p	2.3 e-20
XP_500025	YALI0A13013g	345	n.d.	n.d.	Yps1p	4.2 e-20
XP_500538	YALI0B05654g	397	Ala-17	n.d.	Yps3p	3.9 e-29
XP_002143030	YALI0C10135g	411	Ala-13	n.d.	Yps3p	6.9 e-32
XP_502934	YALI0D17270g	391	Ala-14	n.d.	Yps1p	2.7 e-19
XP_499885	YALI0A08800g	370	Ala-14	n.d.	Yps1p	1.8 e-16
XP_502678	YALI0D10967g	403	Ala-17	n.d.	Pep4p	5.4 e-22
XP_505194	YALI0F09163g	388	Gln-23	n.d.	Yps3p	4.5 e-21
XP_503832	YALI0E11715g	393	Ala-18	n.d.	Bar1p	1.8 e-23
XP_505932	YALI0F27071g	396	Ala-18	n.d.	Pep4p	1.4 e-127
XP_502308	YALI0D02024g	625	Ala-17	n.d.	Pep4p	1.1 e-31
XP_504376	YALI0E24981g	650	n.d.	n.d.	Yps7p	3.8 e-20
XP_504492	YALI0E28006g	203	n.d.	n.d.	Yps1p	2.9 e-27

[a]Putative signal peptide cleavage sites were predicted using SignalP 3.0 (Bendtsen *et al.* 2004).
[b]Putative GPI anchor attachment (omega) sites were predicted using the Big-PI Predictor (Eisenhaber *et al.* 1999).
[c]Blastp searches were performed at the SGD website (yeastgenome.org). The presented e-values reflect homology to the closest *S. cerevisiae* protein sequence.
